# Supplementary material for: A Facile Strategy for Fabrication Lysozyme-Loaded Mesoporous Silica Nanotubes from Electrospun Silk Fibroin Nanofiber Templates
Source: Molecules. 2021 Feb 18;26(4):1073. doi: 10.3390/molecules26041073 (PMC7923156; doi:10.3390/molecules26041073)
Supplement: Supplementary file 1 [file molecules-26-01073-s001.zip › Supporting information/molecules-1088221-supplementary-conversion.docx]

Supplementary Materials

A Facile Strategy for Fabrication Lysozyme-Loaded
Mesoporous Silica Nanotubes from Electrospun Silk Fibroin Nanofiber Templates

Jingxin Zhu ^1,^*, Haijuan Wu ^1^, Ding Wang ^2^, Yanlong Ma ^1^ and Lan Jia ^1^

^1^ College of Materials Science and Engineering, Taiyuan University of Technology, Taiyuan, China; wuhaijuan0093@link.tyut.edu.cn (H.W.); mayanlong@tyut.edu.cn (Y.M.); jialan@tyut.edu.cn (L.J.)

^2^ BOE Photoelectricity Technology Co., Ltd., Chengdu, China; wangdingtyut@126.com

***** Correspondence: zhujingxin@tyut.edu.cn; Tel.:13934526426

1. SEM Images of Silk Nanofibrils in SF/CaCl_2_/FA Solution with Varied CaCl_2_ Concentrations

The degummed silk could be peeled off into silk nanofibrils by CaCl_2_/FA solvent and the size and the diameter of silk nanofibrils were related to the concentration of CaCl_2_ in SF/CaCl_2_/FA solution [1]. Figure S1 shows SEM images of silk nanofibrils in SF/CaCl_2_/FA solution with varied CaCl_2_ concentrations when SF concentration was 15(*w/v*) %. It can be seen that the sizes and the diameters of silk nanofibrils decreased when the concentration of CaCl_2_ in CaCl_2_/FA solvent increased from 3 to 8 (*w/v*) %. The reason may be that with increase concentration of CaCl_2_ in solvent, the ability of CaCl_2_/FA solvent to peel silk was enhanced, making silk filaments thinned and the size and the diameter of silk nanofibrils decreased.





**Figure 1.** SEM images of silk nanofibrils in SF/CaCl_2_/FA solution with varied CaCl_2_ concentrations when SF concentration was 15 (*w/*v) %, (**a,b,c**) was corresponding the concentration of CaCl_2_ in CaCl_2_/FA solvent was 3, 5 and 8 (*w/v*) % respectively; (**a’,b’,c’** ) was local magnification images of (**a,b,c**), respectively.

2. In vitro Release Study

The standard curve of lysozyme is shown in Figure S2, which illustrated the concentration of lysozyme and absorbance have a linear relationship. The standard equation of absorbance and concentration is A = 0.00239C − 0.00648, and the correlation coefficient R^2^ is 0.99493.





**Figure 2.** Standard curve of absorbance-concentration of lysozyme solution.

The cumulative drug release was calculated according to the Equation S1[2].

|  | (S1) |
| --- | --- |

In the Equation S1, E_r_ is the drug release rate, V_e_ is the replacement volume of PBS (2 mL), V_0_ is the total volume of PBS (10 mL), C_i_ is the concentration of lysozyme taken out for the time i (µg/mL), C_n_ is the concentration of lysozyme taken out last time (µg/mL) and m is the total amount of lysozyme in the MSNTs.

Reference

1. Zhang, F.; Lu, Q.; Ming, J.; Dou, H.; Liu, Z.; Zuo, B.; Qin, M.; Li, F.; Kaplan, D.L.; Zhang, X. Silk dissolution and regeneration at the nanofibril scale. *J. Mater. Chem. B* **2014**, *2*, 3879–3885, doi:10.1039/c3tb21582b.

2. Wu, Q.; Tang, X.; Liu, X.; Hou, Y.; Li, H.; Yang, C.; Yi, J.; Song, X.; Zhang, G. Thermo/pH Dual Responsive Mixed-Shell Polymeric Micelles Based on the Complementary Multiple Hydrogen Bonds for Drug Delivery. *Chem. Asian, J.* **2016**, *11*, 112–119.
